# Insulin‐like growth factor‐1 and site‐specific cancers: A Mendelian randomization study

**DOI:** 10.1002/cam4.3345

**Published:** 2020-07-27

**Authors:** Susanna C. Larsson, Paul Carter, Mathew Vithayathil, Siddhartha Kar, Amy M. Mason, Stephen Burgess

**Affiliations:** ^1^ Department of Surgical Sciences Uppsala University Uppsala Sweden; ^2^ Unit of Cardiovascular and Nutritional Epidemiology Institute of Environmental Medicine Karolinska Institutet Stockholm Sweden; ^3^ Department of Public Health and Primary Care University of Cambridge Cambridge UK; ^4^ MRC Cancer Unit University of Cambridge Cambridge UK; ^5^ MRC Integrative Epidemiology Unit Bristol Medical School University of Bristol Bristol UK; ^6^ British Heart Foundation Cardiovascular Epidemiology Unit Department of Public Health and Primary Care University of Cambridge Cambridge UK; ^7^ National Institute for Health Research Cambridge Biomedical Research Centre University of Cambridge and Cambridge University Hospitals Cambridge UK; ^8^ MRC Biostatistics Unit University of Cambridge Cambridge UK

**Keywords:** cancer, insulin‐like growth factor, Mendelian randomization, neoplasm

## Abstract

Insulin‐like growth factor‐1 (IGF‐1) is involved in several processes relevant to carcinogenesis. We used 416 single‐nucleotide polymorphisms robustly associated with serum IGF‐1 levels to assess the potential causal associations between this hormone and site‐specific cancers through Mendelian randomization. Summary‐level genetic association estimates for prostate, breast, ovarian, and lung cancer were obtained from large‐scale consortia including individuals of European‐descent. Furthermore, we estimated genetic associations with 14 site‐specific cancers in European‐descent individuals in UK Biobank. Supplementary analyses were conducted for six site‐specific cancers using summary‐level data from the BioBank Japan Project. Genetically predicted serum IGF‐1 levels were associated with colorectal cancer. The odds ratio (OR) per standard deviation increase of IGF‐1 levels was 1.11 (95% confidence interval [CI] 1.01‐1.22; *P* = .03) in UK Biobank and 1.22 (95% CI 1.09‐1.36; *P* = 3.9 × 10^−4^) in the BioBank Japan Project. For prostate cancer, the corresponding OR was 1.10 (95% CI 1.01‐1.21; *P* = .04) in UK Biobank, 1.03 (95% CI 0.97‐1.09; *P* = .41) in the prostate cancer consortium, and 1.08 (95% CI 0.95‐1.22; *P* = .24) in the BioBank Japan Project. For breast cancer, the corresponding OR was 0.99 (95% CI 0.92‐1.07; *P* = .85) in UK Biobank and 1.08 (95% CI 1.02‐1.13; *P* = 4.4 × 10^−3^) in the Breast Cancer Association Consortium. There was no statistically significant association between genetically predicted IGF‐1 levels and 14 other cancers. This study found some support for a causal association between elevated serum IGF‐1 levels and increased risk of colorectal cancer. There was inconclusive or no evidence of a causal association of IGF‐1 levels with prostate, breast, and other cancers.

## Graphical abstract

This Mendelian randomization study found support for a causal association between elevated serum insulin‐like growth factor‐1 levels and increased risk of colorectal cancer.

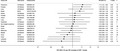

## 1 INTRODUCTION

Insulin‐like growth factor‐1 (IGF‐1) is involved in several processes relevant to carcinogenesis, such as cell proliferation and apoptosis.^1^ Acromegaly, which is characterized by increased growth hormone levels with concomitant raised IGF‐1 levels, is associated with higher risk of cancer.^2^, ^3^ Moreover observational studies in non‐acromegalic populations have reported that IGF‐1 levels are positively associated with risk of colorectal,^4^, ^5^, ^6^ prostate^5^, ^7^ and breast cancer,^5^, ^8^ but inversely with risk of ovarian cancer.^9^ Data on circulating IGF‐1 levels in relation to other cancers are limited and inconclusive.^1^, ^5^ Considering that circulating IGF‐1 levels can be altered by diet (particularly by reduced milk and protein intake^10^, ^11^, ^12^, ^13^, ^14^) and medical therapy, establishing the causal association between circulating IGF‐1 levels and cancer risk are important from a public health and clinical perspective.

Mendelian randomization (MR) is a method to evaluate causality by exploiting genetic variants with a strong association with the exposure (eg IGF‐1 levels) as instrumental variables to predict the effect of the exposure on disease risk.^15^ We used the MR technique to examine the potential causal associations between serum IGF‐1 levels and site‐specific cancers.

## 2 MATERIALS AND METHODS

### 2.1 Genetic instrument

Single‐nucleotide polymorphisms (SNPs) strongly associated with serum IGF‐1 (at *P* < 5 × 10^−8^) were taken from a genome‐wide association study of 358 072 European‐descent participants of UK Biobank.^16^ After omitting correlated SNPs (linkage disequilibrium *R* ^2^ > 0.01), 416 SNPs remained and were used as instrumental variables for IGF‐1 levels (Table S1). The IGF‐1 SNPs have been shown to have clear enrichment of genome‐wide significant signals in core genes and pathways related to growth hormone‐IGF cascade, in particular the upper parts of the cascade that regulate IGF‐1 release, but also downstream components of the cascade suggesting feedback mechanism on IGF‐1 levels.^17^ The variance in IGF‐1 levels explained by the SNPs was 9.4%. The F‐statistic of the genetic instrument was 80.9. In UK Biobank as a whole, serum IGF‐1 concentration had a mean value of 21.4 nmol/L (standard deviation [SD] 5.7 nmol/L) and ranged from 14.2 nmol/L in the first decile to 28.4 nmol/L in the ninth decile.

### 2.2 Data sources for cancer

Publicly available summary statistics estimates for prostate, breast, ovarian, and lung cancer were obtained, respectively, from the Prostate Cancer Association Group to Investigate Cancer Associated Alterations in the Genome (PRACTICAL) consortium,^18^ Breast Cancer Association Consortium (BCAC),^19^ Ovarian Cancer Association Consortium (OCAC),^20^ and International Lung Cancer Consortium (ILCCO).^21^ The consortia included participants of European ancestry only. We additionally estimated genetic associations with site‐specific cancers with at least 1000 cases (n = 14 cancers) among 367 586 unrelated individuals of European ancestry (aged 37‐73 years at baseline) in UK Biobank. The analyses were conducted using logistic regression analysis adjusted for age, sex, and ten genetic principal components. Cancer ascertainment data was until 31 March 2017, and outcomes were obtained from the national cancer registry, electronic health records, hospital episode statistics data, death certification data, and self‐reported information validated by nurse interview (Table S2). Associations with prostate cancer were estimated in men only (n = 168 748) and associations with breast, ovarian, uterine, and cervical cancer in women only (n = 198 838). UK Biobank was not included in any of the consortia. In a supplementary analysis, we used summary statistics data for cancer sites with at least 1000 cases (breast cancer data were not available) from the BioBank Japan Project.^22^ Ethical approval to conduct this MR analysis based on summary statistics and UK Biobank data had been obtained from the Swedish Ethical Review Authority.

### 2.3 Statistical analysis

The multiplicative random‐effects inverse variance weighted method was applied for the main analyses.^23^ Heterogeneity among estimates derived from individual SNPs was assessed using the I^2^ statistic.^24^ We have shown that genetically predicted IGF‐1 levels are robustly related to adult height but not adiposity traits.^25^ To evaluate the direct effect of any observed association between IGF‐1 levels and cancer not mediated via height we used multivariable MR analysis.^26^ Summary statistics estimates for height were obtained from UK Biobank (analyses by Neale Lab) via the MR‐Base platform (http://www.mrbase.org/). As sensitivity analyses, we used the weighted median, MR‐Egger, MR Pleiotropy RESidual Sum and Outlier (MR‐PRESSO) and contamination mixture methods.^23^, ^27^, ^28^, ^29^ In an additional sensitivity analysis, we excluded self‐reported cancer in UK Biobank. We further conducted a sensitivity analysis using the SNP in the *IGF1* gene (rs11111274), which was strongly associated with IGF‐1 levels (7.59 × 10^−175^), as genetic instrument. This SNP was not available in the PRACTICAL consortium, BCAC, and OCAC, but a proxy SNP (rs1520222, in complete linkage disequilibrium with rs11111274) was used in the analysis based on the PRACTICAL consortium. No suitable proxy SNP (linkage disequilibrium *R* ^2^ > .8) was available in the BCAC and OCAC. This variant only explains 0.2% of the variance in serum IGF‐1. However, as the *IGF1* locus is the coding region for IGF‐1, this analysis has particular biological relevance for IGF‐1. The analyses were carried out using the mrrobust,^30^ MendelianRandomization,^31^ and MR‐PRESSO^28^ packages. All odds ratios (OR) were expressed per 1 SD (about 5.7 nmol/L) increment in IGF‐1 levels.

We estimated the power for different cancers using a web tool.^32^ Associations with *P* values below the Bonferroni‐corrected threshold of <0.0036 (0.05/14 site‐specific cancers in European‐descent individuals) were deemed strong evidence of association, whereas those with *P* values ranging from .0036 and .05 were regarded as suggestive support for a possible association.

### 2.4 Data availability

Summary‐level data from the PRACTICAL consortium,^18^ BCAC,^19^ OCAC,^20^ ILCCO,^21^ and BBJ^22^ are publicly available. Data from the UK Biobank study are accessible upon application (https://www.ukbiobank.ac.uk/).

## 3 RESULTS

### 3.1 Statistical power

The power in the analyses of different cancer sites are shown in Table S3. We had 80% to 100% power to detect an OR of 1.10 (or 0.90) in analyses of cancers of the prostate and breast as well as in analyses of ovarian cancer in OCAC. The power was 80% or higher at ORs of 1.20 and 1.30 in analyses of cancers with 2000 and 1000 cases, respectively, in UK Biobank and the BioBank Japan Project.

### 3.2 IGF‐1 and cancer in European‐descent individuals

**FIGURE 1:**
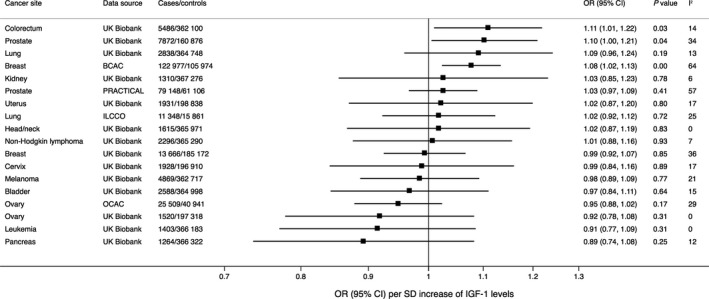
Associations of genetically predicted serum insulin‐like growth factor‐1 (IGF‐1) levels with site‐specific cancers in European‐descent individuals. Estimates were derived using the multiplicative random‐effects inverse‐variance weighted method and were based on up to 416 single‐nucleotide polymorphisms associated with IGF‐1 levels at the genome‐wide significance threshold. The I^2^ value is a measure of heterogeneity among estimates from individual single‐nucleotide polymorphisms. BCAC, Breast Cancer Association Consortium; ILCCO, International Lung Cancer Consortium; OCAC, Ovarian Cancer Association Consortium; PRACTICAL, Prostate Cancer Association Group to Investigate Cancer Associated Alterations in the Genome

The associations of genetic predisposition to higher serum levels of IGF‐1 with the 14 site‐specific cancers in European‐descent individuals based on consortia and UK Biobank data are shown in Figure 1. There was a suggestive positive association of genetically predicted serum IGF‐1 levels with colorectal cancer. The OR per SD increase of genetically predicted IGF‐1 levels was 1.11 (95% confidence interval [CI], 1.01‐1.22; *P* = .03). For prostate cancer, the corresponding OR was 1.10 (95% CI 1.01‐1.21; *P* = .04) in UK Biobank and 1.03 (95% CI 0.97‐1.09; *P* = .41) in the PRACTICAL consortium. Genetically predicted higher IGF‐1 levels was significantly associated with higher odds of breast cancer in the BCAC (OR, 1.08; 95% CI 1.02‐1.13; *P* = 4.4 × 10^−3^), with similar estimates for estrogen‐receptor positive (OR, 1.07; 95% CI 1.01‐1.13; *P* = .02) and estrogen‐receptor negative (OR, 1.08; 95% CI 1.00‐1.16; *P* = .04) breast tumors. However, there was no association between genetically predicted IGF‐1 levels and breast cancer in UK Biobank (OR, 0.99; 95% CI 0.92‐1.07; *P* = .85). The associations of genetically predicted IGF‐1 levels with cancers of the colorectum, prostate, and breast were similar after adjustment for height through multivariable MR analysis (Table S4). There was weak or no evidence of association between IGF‐1 levels and 11 other cancers (Figure 1).

Results were consistent in sensitivity analysis, except for suggestive evidence of a positive association between genetically predicted IGF‐1 levels and lung cancer in UK Biobank in the MR‐Egger analysis (Table S5). However, no association of IGF‐1 levels with lung cancer was observed in other sensitivity analyses or in analyses based on the ILCCO dataset (Table S5). Similar results were observed when excluding self‐reported cancer in UK Biobank (Figure S1). For example, the ORs per SD increase of genetically predicted IGF‐1 levels were 1.13 (95% CI 1.03‐1.25; *P* = .01) for colorectal cancer and 1.10 (95% CI 1.00‐1.21; *P* = .05) for prostate cancer.

In analysis using the SNP in the *IGF1* gene as instrument, a suggestive association between genetically predicted IGF‐1 levels and colorectal cancer was observed (OR 1.74; 95% CI 1.02‐2.97; *P* = .04). There was also a suggestive association between genetically predicted IGF‐1 levels instrumented by the SNP in the *IGF1* gene and prostate cancer, both in the PRACTICAL consortium (OR 1.33; 95% CI 1.02‐1.65; *P* = .01) and UK Biobank (OR 1.67; 95% CI 1.05‐2.65; *P* = .03). The SNP in the *IGF1* gene was not associated with the other site‐specific cancers in UK Biobank (*P* > .05).

### 3.3 IGF‐1 and cancer in Japanese individuals

**FIGURE 2:**
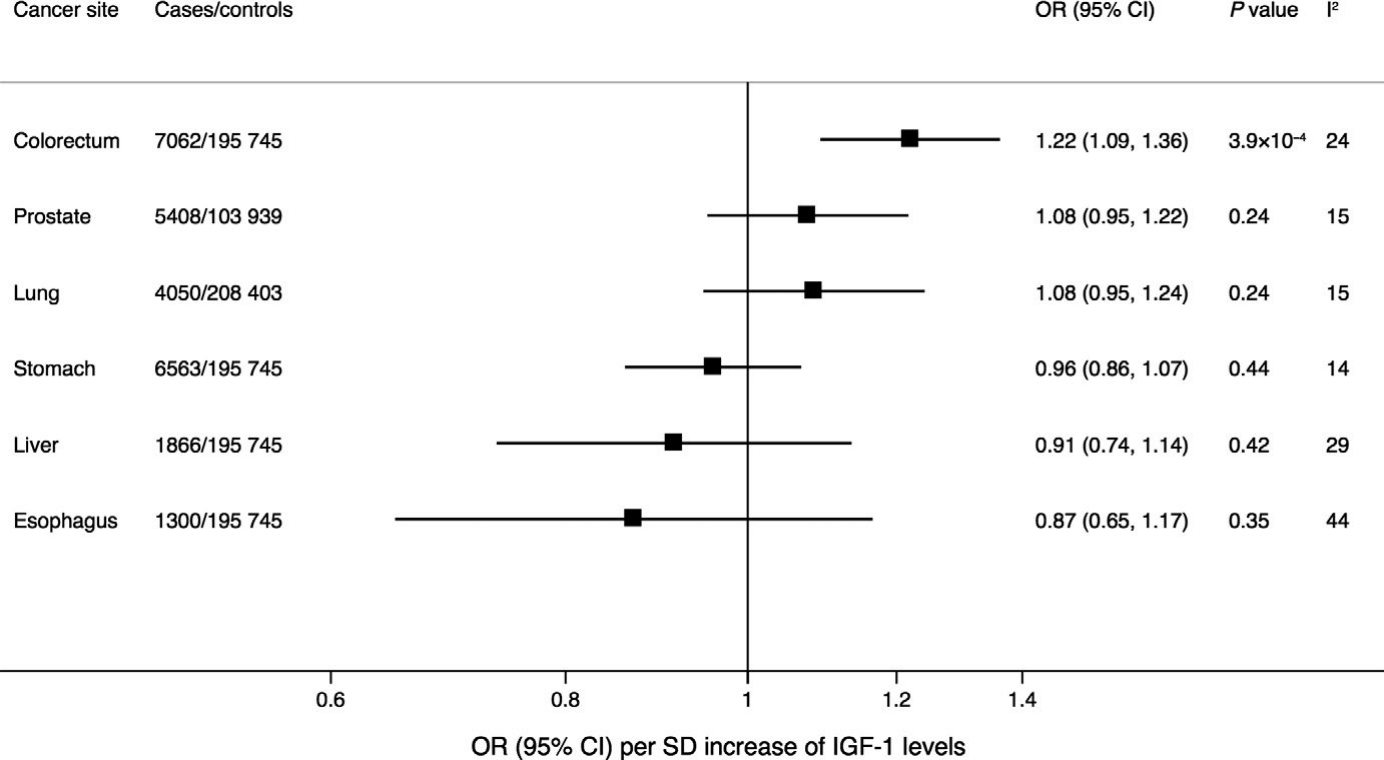
Associations of genetically predicted serum insulin‐like growth factor‐1 (IGF‐1) levels with site‐specific cancers in Japanese individuals. Estimates were derived using the multiplicative random‐effects inverse‐variance weighted method and were based on 336 single‐nucleotide polymorphisms associated with IGF‐1 levels at the genome‐wide significance threshold and available in the BioBank Japan Project genome‐wide‐association study.^22^ The I^2^ value is a measure of heterogeneity among estimates from individual single‐nucleotide polymorphisms

In a supplementary analysis using data from a genome‐wide association study in a Japanese population, genetically predicted IGF‐1 levels were statistically significantly associated with colorectal cancer but not prostate, lung, esophageal, stomach, or liver cancer (Figure 2). The OR of colorectal cancer per SD increase of genetically predicted IGF‐1 levels was 1.22 (95% CI 1.09‐1.36; *P* = 3.9 × 10^−4^) when using the full set of SNPs (Figure 2) and 1.57 (95% CI 1.02‐2.41; *P* = .04) when using the SNP in the *IGF1* gene. The corresponding ORs of prostate cancer were 1.08 (95% CI 0.95‐1.22; *P* = .24) (Figure 2) and 0.99 (95% CI 0.60‐1.65; *P* = .98), respectively.

## 4 DISCUSSION

In this MR analysis assessing the potential causal relation between serum IGF‐1 levels and several cancers, we found some evidence that increased IGF‐1 levels may increase the risk of colorectal cancer. Findings for genetically predicted IGF‐1 levels in relation to prostate and breast cancer were inconsistent with a suggestive positive association observed only in UK Biobank (for prostate cancer) or the BCAC (for breast cancer), or when using the SNP in the *IGF1* gene as instrument (for prostate cancer).

Our finding for colorectal cancer confirm those of a previous MR study which showed an OR of colorectal cancer of 1.08 (95% CI 1.03‐1.12) per one SD increment of genetically‐predicted IGF‐1 levels.^6^ That study further showed that directly measured serum IGF‐1 levels were associated with increased risk of all colorectal tumor subsites, including proximal colon, distal colon, and rectal cancer.^6^ Possible mechanisms underlying the increased colorectal cancer risk include direct actions of IGF‐1 on cell growth or indirect effects via for example insulin resistance and elevated insulin levels.^25^

The present MR results for IGF‐1 and breast cancer based on BCAC data are similar to those obtained from another MR study also based on BCAC data which showed an OR of 1.05 (95% CI 1.01‐1.10) per 5 nmol/L increase of genetically predicted IGF‐1 levels based on 265 SNPs associated with serum IGF‐1 levels in women.^33^ Another smaller case‐control study (4647 cases and 4564 controls) found that the IGF‐1‐increasing allele of rs1520220 in the *IGF1* gene was associated with higher odds of breast cancer.^34^ We could not replicate an association between genetically predicted IGF‐1 levels and breast cancer in UK Biobank, potentially owing to the lack of power to detect a weak association (41% and 93% power to detect an OR of 1.05 and 1.10, respectively), phenotyping differences, or younger participants in UK Biobank (eg some current controls in UK Biobank may eventually develop cancer later in their life). Given these conflicting results, further very large MR studies of IGF‐1 levels and breast cancer are needed to determine whether there is a causal relationship between elevated IGF‐1 levels and breast cancer risk.

Results of meta‐analyses of observational studies have revealed that high IGF‐1 levels are associated with an increased risk of prostate cancer^5^, ^7^ and with a lower ovarian cancer risk.^9^ In this MR study of genetically predicted IGF‐1 levels and risk of prostate and ovarian cancer, results were in the same direction as those from observational studies but were not statistically significant. However, we found a suggestive positive association of genetically predicted IGF‐1 levels with prostate cancer in UK Biobank (OR 1.10; *P* = .04) and a similar non‐significant estimate in the Biobank Japanese Project (OR 1.08; *P* = .24). The reason for the inconsistent results might be related to phenotyping differences and different proportions of advanced stage prostate cancer cases across datasets.

A strength of this study is the use of several data sources of large sample sizes. Moreover we confined the analyses to major cancers with at least 1000 cases to ensure sufficient power. A limitation is that the genetic associations with IGF‐1 levels were estimated in UK Biobank from which we also obtained genetic association estimates for cancer. This may have affected the results in the direction of the observational association in the analyses of UK Biobank data. Nevertheless, given the strong genetic instrument for IGF‐1 levels (*F* statistic > 10), bias from sample overlap would be small.^35^ Moreover, the association between genetically predicted IGF‐1 levels and colorectal cancer was replicated in an independent cohort of Japanese individuals. These supplementary results based on data from the Biobank Japan Project should nevertheless be interpreted with caution as the genetic variants for serum IGF‐1 levels were identified in a European population and was not verified in a Japanese population. Future investigations using Asian‐specific genome‐wide association study estimates for IGF‐1 are required to obtain more accurate causal estimates for IGF‐1 and cancer in Asians. Another limitation is that we had insufficient power to assess potential non‐linear relationships between IGF‐1 levels and cancer. A further shortcoming is that data on advanced stage prostate cancer were not available. Hence, more research is necessary to clarify whether IGF‐1 plays a role in the development of advanced stage prostate cancer.

In conclusion, these MR findings support a potential causal association between increased serum IGF‐1 levels and higher risk of colorectal cancer. There was inconclusive evidence of an association of serum IGF‐1 levels with prostate and breast cancer, and weak or no evidence for an association with other major cancers. These results advocate the current clinical practice for colorectal cancer surveillance in patients with acromegaly and elevated serum IGF‐1 levels.^36^

## CONFLICT OF INTEREST

The authors declare no conflict of interest.

## AUTHOR CONTRIBUTIONS

Susanna C. Larsson contributed to conceptualization, methodology, data acquisition and curation, formal analysis, visualization, writing, and editing. Paul Carter, Mathew Vithayathil, Siddhartha Kar, and Amy M. Mason contributed to interpretation of data, writing, and editing. Stephen Burgess contributed to methodology, interpretation of data, writing, and editing. All the authors gave final approval of the version.

## Supporting information

Supplementary Material

## Data Availability

Data used in this study are publicly available. Data from the UK Biobank study are accessible upon application (https://www.ukbiobank.ac.uk/).
