# Supplementary material for: Insulin‐like growth factor‐1 and site‐specific cancers: A Mendelian randomization study
Source: Cancer Med. 2020 Jul 27;9(18):6836–42. doi: 10.1002/cam4.3345 (PMC7520358; doi:10.1002/cam4.3345)
Supplement: Supplementary file 1 — Supplementary Material [file CAM4-9-6836-s001.pdf]

Table S1. Single-nucleotide polymorphisms associated with insulin-like growth factor-1

SNP	Chr	EA	NEA	Beta	SE	*P* value	Nearby gene
rs10159299	1	C	T	0,0176	0,0025	5.112e-12	PLXNA2
rs1046011	1	T	C	0,0209	0,0027	3.303e-14	LEPROT
rs10779509	1	C	T	0,0144	0,0026	2.109e-08	RP1-272L16.1
rs11165778	1	A	G	0,0159	0,0028	1.492e-08	HFM1
rs112436634	1	T	C	0,0161	0,0027	1.749e-09	PEX14
rs1127313	1	G	A	0,0240	0,0025	1.394e-21	ADAR
rs11577063	1	T	G	0,0203	0,0030	1.511e-11	AXDND1
rs12141189	1	T	C	0,0449	0,0029	4.135e-53	HLX
rs1223763	1	T	G	0,0238	0,0033	8.461e-13	RP11-53A1.3
rs12723255	1	C	T	0,0170	0,0026	3.229e-11	EIF4G3
rs12749024	1	T	C	0,0755	0,0036	7.72e-100	PAPPA2
rs140604451	1	G	A	0,0540	0,0082	3.524e-11	GSTM2
rs1430753	1	A	G	0,0210	0,0032	7.085e-11	WLS
rs143885630	1	G	A	0,0303	0,0038	1.358e-15	SMG7
rs165316	1	G	A	0,0731	0,0032	9.66e-119	RPL5P6
rs17037452	1	A	G	0,0228	0,0034	1.658e-11	CLCN6
rs17393144	1	A	G	0,0155	0,0027	9.319e-09	MIR34AHG
rs1825813	1	A	G	0,0231	0,0031	1.879e-13	C1orf146
rs2075995	1	C	A	0,0144	0,0025	9.790e-09	E2F2
rs2724373	1	C	T	0,0186	0,0026	1.436e-12	C1orf132
rs2802330	1	G	A	0,0310	0,0033	4.632e-21	PDIK1L
rs2802951	1	A	G	0,0163	0,0028	4.970e-09	RN7SL668P
rs2819336	1	T	C	0,0273	0,0026	1.550e-25	PTPRF
rs3131646	1	G	T	0,0155	0,0027	1.710e-08	MYCL
rs36086195	1	C	T	0,0189	0,0025	1.359e-13	ARHGEF19-AS1
rs4306136	1	A	G	0,0173	0,0026	1.997e-11	RP11-103C3.1

rs569356	1	G	A	0,0271	0,0036	5.953e-14	OPRD1
rs599839	1	G	A	0,0314	0,0030	9.629e-26	CELSR2
rs61780439	1	G	A	0,0209	0,0030	5.323e-12	SLFNL1
rs6659176	1	C	G	0,0419	0,0046	1.354e-19	NR0B2
rs6701954	1	T	G	0,0140	0,0025	3.497e-08	USP48
rs684818	1	T	C	0,0236	0,0025	9.658e-21	RP4-781K5.7
rs708108	1	T	C	0,0149	0,0026	5.872e-09	WNT3A
rs7517340	1	C	T	0,0347	0,0033	2.123e-26	AKT3
rs7528548	1	C	T	0,0543	0,0088	7.954e-10	PYGO2
rs7539178	1	C	A	0,0263	0,0037	1.365e-12	JAK1
rs7545345	1	C	T	0,0265	0,0037	1.032e-12	NUCKS1
rs75681856	1	T	C	0,0232	0,0040	5.171e-09	RABGAP1L
rs75907879	1	T	C	0,0243	0,0038	1.425e-10	SPEN
rs77369503	1	G	A	0,0452	0,0070	1.397e-10	RGS4
rs903908	1	C	T	0,0160	0,0025	2.176e-10	SKI
rs11677980	2	G	A	0,0151	0,0027	3.043e-08	LBH
rs11678946	2	A	C	0,0140	0,0025	3.143e-08	EPHA4
rs12471768	2	C	T	0,0220	0,0028	1.639e-15	SERTAD2
rs1260326	2	C	T	0,0632	0,0026	9.66e-133	GCKR
rs12710648	2	A	G	0,0174	0,0025	5.703e-12	SMC6
rs1465529	2	T	C	0,0194	0,0027	1.386e-12	SP110
rs17050272	2	G	A	0,0235	0,0026	3.201e-20	AC073257.2
rs17323117	2	G	A	0,0287	0,0047	7.980e-10	NEU2
rs17400325	2	C	T	0,0539	0,0065	9.393e-17	PDE11A
rs2674492	2	A	G	0,0141	0,0026	4.835e-08	CYBRD1
rs35135518	2	T	C	0,0286	0,0040	1.521e-12	AC010145.4
rs35641591	2	C	T	0,0496	0,0065	1.575e-14	PCBP1-AS1
rs3791679	2	G	A	0,0176	0,0030	4.338e-09	EFEMP1
rs4402747	2	A	G	0,0159	0,0025	2.960e-10	NEU2

rs58387407	2	G	A	0,0175	0,0032	3.502e-08	CACNB4
rs62136965	2	C	T	0,0370	0,0060	6.429e-10	RNU6-566P
rs62182127	2	A	G	0,0193	0,0025	2.712e-14	VIL1
rs6435156	2	C	T	0,0244	0,0029	2.045e-17	BMPR2
rs6437249	2	C	T	0,0188	0,0027	4.559e-12	HDLBP
rs6544549	2	T	C	0,0240	0,0037	1.592e-10	KCNG3
rs6749680	2	A	G	0,0148	0,0026	9.868e-09	ALMS1
rs6760135	2	T	C	0,0502	0,0030	1.194e-63	ASXL2
rs702878	2	A	G	0,0143	0,0025	1.815e-08	AC074391.1
rs73954943	2	A	G	0,0313	0,0051	1.063e-09	BCL2L11
rs7574340	2	T	C	0,0172	0,0027	2.971e-10	SLC8A1
rs7578633	2	T	C	0,0182	0,0026	3.379e-12	PAX8
rs112893170	3	T	C	0,0204	0,0032	1.527e-10	FEZF2
rs11717397	3	G	A	0,0150	0,0025	2.572e-09	UBE2E2
rs11928797	3	A	C	0,0301	0,0039	2.428e-14	UBP1
rs12491473	3	G	A	0,0199	0,0025	5.913e-15	CCDC12
rs13069961	3	G	A	0,0184	0,0031	3.680e-09	KALRN
rs13073970	3	T	G	0,0246	0,0031	2.016e-15	EIF5A2
rs1822825	3	G	A	0,0139	0,0025	2.886e-08	PPARG
rs2268829	3	G	A	0,0182	0,0031	3.038e-09	DGKG
rs2607748	3	C	T	0,0168	0,0025	3.289e-11	CHCHD4
rs3772102	3	G	T	0,0204	0,0025	5.954e-16	ST3GAL6
rs4678497	3	C	T	0,0165	0,0026	1.959e-10	SUSD5
rs504603	3	C	T	0,0291	0,0049	2.402e-09	BZW1P1
rs55717031	3	G	T	0,0319	0,0027	2.474e-31	MRPS22
rs56062334	3	T	C	0,0172	0,0026	1.839e-11	LINC02068
rs62263345	3	A	G	0,0281	0,0037	1.640e-14	BBX
rs62280667	3	C	T	0,0282	0,0027	2.477e-26	SENP7
rs6440008	3	T	C	0,0352	0,0026	1.113e-41	ZBTB38

rs66707192	3	G	A	0,0182	0,0029	4.415e-10	HRG
rs687339	3	T	C	0,0399	0,0030	2.666e-40	KRT18P35
rs73238159	3	C	T	0,0254	0,0037	1.059e-11	XRN1
rs7625680	3	A	G	0,0151	0,0026	7.693e-09	ATG7
rs7628689	3	G	A	0,0288	0,0035	6.825e-17	C3orf38
rs811332	3	C	T	0,0195	0,0032	2.011e-09	MRAS
rs9819762	3	T	C	0,0185	0,0033	1.212e-08	PIK3CA
rs1055582	4	C	T	0,0274	0,0025	1.578e-27	UBE2K
rs111443396	4	C	T	0,0255	0,0040	2.015e-10	LINC01091
rs1229984	4	T	C	0,1044	0,0085	1.219e-34	ADH1B
rs13108218	4	G	A	0,0171	0,0026	5.920e-11	HGFAC
rs17429745	4	G	T	0,0260	0,0027	2.045e-21	RP11-556I14.1
rs1902023	4	A	C	0,0251	0,0025	1.951e-23	UGT2B15
rs2280099	4	G	A	0,0250	0,0033	1.923e-14	TIGD2
rs35036084	4	T	C	0,0167	0,0026	8.218e-11	RP11-145G20.1
rs3804173	4	G	A	0,0196	0,0027	4.484e-13	PRDM5
rs4394044	4	T	C	0,0140	0,0026	4.708e-08	SORBS2
rs62302688	4	G	A	0,0394	0,0043	4.057e-20	GABRA2
rs62334147	4	C	T	0,0192	0,0033	4.261e-09	DDX60L
rs62342064	4	C	T	0,0222	0,0037	1.836e-09	RP11-119H12.3
rs6532798	4	T	C	0,0370	0,0027	1.364e-41	ADH4
rs6827641	4	T	C	0,0140	0,0025	2.623e-08	HHIP
rs6853741	4	A	G	0,0237	0,0029	1.676e-16	ARHGAP10
rs7667562	4	C	A	0,0165	0,0028	3.770e-09	LARP1B
rs976002	4	A	G	0,0360	0,0029	4.672e-35	TMPRSS11E
rs11242236	5	G	A	0,0246	0,0025	1.957e-22	C5orf66
rs11954036	5	T	C	0,0375	0,0027	1.936e-44	PDE4D
rs12108803	5	G	T	0,0331	0,0058	1.015e-08	TBCA
rs12520263	5	T	G	0,0169	0,0028	2.579e-09	RP11-357F12.1

rs13168379	5	A	G	0,0306	0,0049	6.148e-10	CPEB4
rs13178887	5	T	C	0,0231	0,0026	2.712e-19	MEF2C-AS1
rs1498603	5	T	G	0,0311	0,0051	1.170e-09	PDE4D
rs17714046	5	C	T	0,0424	0,0060	1.197e-12	TRIM41
rs2042253	5	T	C	0,0227	0,0029	1.017e-14	MIR5197
rs2227819	5	T	C	0,0218	0,0040	4.322e-08	F2R
rs2366398	5	T	G	0,0185	0,0031	1.613e-09	CTD-2151A2.3
rs258775	5	A	C	0,0252	0,0033	3.394e-14	ARHGAP26
rs26822	5	G	A	0,0174	0,0027	9.845e-11	PPIP5K2
rs28650790	5	T	C	0,0175	0,0032	4.108e-08	C5orf67
rs329122	5	A	G	0,0181	0,0025	1.312e-12	JADE2
rs35668185	5	T	C	0,0561	0,0031	3.402e-73	SLIT3
rs3734166	5	A	G	0,0277	0,0029	8.063e-22	CDC25C
rs6180	5	A	C	0,0347	0,0025	6.019e-43	GHR
rs6895953	5	G	A	0,0240	0,0025	5.167e-21	RP11-357F12.1
rs72758321	5	G	A	0,0470	0,0060	3.926e-15	PLCXD3
rs73271090	5	G	A	0,0440	0,0034	7.844e-39	CTB-1I21.1
rs7719168	5	C	A	0,0303	0,0040	2.429e-14	15ARL
rs80170948	5	G	T	0,0385	0,0064	2.221e-09	SREK1IP1
rs840809	5	A	C	0,0162	0,0028	1.183e-08	CTD-2232E5.2
rs9292578	5	C	A	0,0396	0,0063	3.195e-10	PRLR
rs1042335	6	T	C	0,0151	0,0027	3.568e-08	HLA-DPB1
rs1130838	6	C	T	0,0264	0,0026	1.848e-23	HLA-C
rs113127944	6	A	G	0,0508	0,0078	6.115e-11	CENPW
rs1150781	6	G	C	0,0263	0,0044	2.337e-09	C6orf1
rs1165196	6	G	A	0,0289	0,0025	6.414e-30	SLC17A1
rs12110787	6	A	C	0,0225	0,0040	1.857e-08	MAP3K4
rs12194618	6	A	G	0,0171	0,0026	2.686e-11	ZFAND3
rs2296198	6	C	T	0,0163	0,0029	1.937e-08	RNF144B

rs2397112	6	A	G	0,0185	0,0025	3.313e-13	RP11-228O6.2
rs28362677	6	T	C	0,0318	0,0035	1.763e-19	BTNL2
rs3008051	6	C	T	0,0144	0,0026	1.946e-08	PDE10A
rs3127579	6	A	G	0,0326	0,0036	1.230e-19	SLC22A2
rs3890746	6	C	T	0,0204	0,0025	1.060e-15	L3MBTL3
rs41285260	6	T	G	0,0385	0,0045	5.123e-18	CENPW
rs4709995	6	T	C	0,0422	0,0026	7.369e-60	SDIM1
rs584955	6	A	G	0,0362	0,0061	2.579e-09	TMEM14C
rs670049	6	A	C	0,0192	0,0027	6.417e-13	Y_RNA
rs6916994	6	C	T	0,0294	0,0025	1.519e-31	GJB7
rs6924225	6	G	A	0,0193	0,0034	1.442e-08	RUNX2
rs73382439	6	C	T	0,0188	0,0033	1.800e-08	E2F3
rs7740433	6	A	G	0,0167	0,0029	1.504e-08	CNPY3
rs7758644	6	A	C	0,0192	0,0034	1.514e-08	RP1-155D22.1
rs7774230	6	T	C	0,0260	0,0025	5.464e-25	ESR1
rs790513	6	C	A	0,0253	0,0029	1.575e-18	OPRM1
rs9321106	6	A	G	0,0184	0,0033	2.408e-08	PTPRK
rs9322822	6	C	T	0,0153	0,0027	1.349e-08	LIN28B-AS1
rs9364815	6	A	G	0,0148	0,0027	2.829e-08	PDE10A
rs9398171	6	T	C	0,0504	0,0028	9.510e-74	FOXO3
rs9398891	6	T	C	0,0175	0,0027	1.108e-10	LAMA2
rs998584	6	A	C	0,0202	0,0025	1.211e-15	VEGFA
rs10246481	7	G	A	0,0147	0,0026	1.499e-08	AC073133.2
rs10252510	7	G	A	0,0203	0,0027	8.179e-14	GHRHR
rs1050327	7	A	G	0,0168	0,0025	2.907e-11	ZMIZ2
rs114949263	7	T	C	0,0271	0,0040	1.283e-11	TMEM176B
rs11556924	7	C	T	0,0158	0,0026	9.798e-10	ZC3HC1
rs1182174	7	A	G	0,0208	0,0028	4.808e-14	GNA12
rs12666306	7	G	A	0,0170	0,0025	1.702e-11	RP11-222O23.1

rs12699547	7	C	T	0,0213	0,0026	2.438e-16	MAD1L1
rs145188037	7	A	G	0,1203	0,0111	1.749e-27	IGFBP3
rs17145738	7	T	C	0,0338	0,0038	8.750e-19	TBL2
rs1986692	7	G	A	0,0150	0,0026	8.640e-09	EXOC4
rs2048672	7	C	A	0,0181	0,0028	6.405e-11	LINC-PINT
rs207212	7	C	T	0,0280	0,0042	1.659e-11	LINC00513
rs2228078	7	C	T	0,0569	0,0104	4.076e-08	GHRHR
rs2250243	7	C	T	0,0236	0,0030	1.694e-15	ZNF316
rs2270628	7	T	C	0,0329	0,0032	3.737e-25	IGFBP3
rs273956	7	A	G	0,0207	0,0025	5.369e-16	CREB3L2
rs2896395	7	C	T	0,0154	0,0028	2.446e-08	SND1
rs34312198	7	A	C	0,0241	0,0040	1.345e-09	ZNF3
rs35862187	7	A	G	0,0311	0,0056	3.484e-08	AUTS2
rs411717	7	T	C	0,0152	0,0025	2.144e-09	COL1A2
rs4719393	7	T	G	0,0268	0,0027	1.290e-22	DGKB
rs74657816	7	T	G	0,0473	0,0054	3.006e-18	HMGN1P19
rs7783012	7	G	A	0,0164	0,0026	1.561e-10	FOXP2
rs7802508	7	A	G	0,0212	0,0026	9.477e-17	ZFAND2A
rs79881512	7	C	T	0,0587	0,0107	4.185e-08	AC073325.1
rs870796	7	G	A	0,0168	0,0025	3.274e-11	ELK1P1
rs11782452	8	G	A	0,0146	0,0026	1.258e-08	BNIP3L
rs12549853	8	A	G	0,0155	0,0026	1.654e-09	PLEC
rs1431015	8	C	T	0,0195	0,0026	2.655e-14	RNU2-54P
rs1495741	8	G	A	0,0262	0,0030	5.034e-18	NAT2
rs1786342	8	T	C	0,0174	0,0026	1.241e-11	SNX31
rs2737205	8	T	C	0,0233	0,0025	4.711e-20	TRPS1
rs2978062	8	G	T	0,0188	0,0034	3.225e-08	ST3GAL1
rs445036	8	T	C	0,0191	0,0028	5.794e-12	ZBTB10
rs56352849	8	A	G	0,0158	0,0028	1.497e-08	KCNB2

rs60862542	8	G	A	0,0172	0,0031	1.648e-08	EIF3E
rs6473015	8	C	A	0,0194	0,0028	2.994e-12	AC105242.1
rs716100	8	A	G	0,0194	0,0027	7.196e-13	ZFAT
rs76393968	8	G	A	0,0596	0,0100	2.566e-09	MSR1
rs9657541	8	C	T	0,0196	0,0031	3.846e-10	CTD-2135J3.4
rs1055710	9	G	A	0,0183	0,0027	7.149e-12	FAM120AOS
rs10757291	9	G	A	0,0194	0,0025	1.487e-14	CDKN2B-AS1
rs10811787	9	C	T	0,0145	0,0025	7.833e-09	RP11-370B11.4
rs10869022	9	C	T	0,0213	0,0032	1.633e-11	TRPM3
rs10908903	9	T	G	0,0154	0,0025	1.288e-09	GADD45G
rs11557154	9	T	C	0,0237	0,0038	3.100e-10	DCAF12
rs13301073	9	G	A	0,0219	0,0026	4.056e-17	MAPKAP1
rs2378662	9	G	A	0,0167	0,0025	4.195e-11	RP11-158D2.2
rs28831479	9	C	A	0,0222	0,0029	1.196e-14	PTCH1
rs7034716	9	C	T	0,0153	0,0028	2.595e-08	TGFBR1
rs7041137	9	T	C	0,0171	0,0028	7.298e-10	RAD23B
rs7872812	9	T	C	0,0259	0,0036	3.162e-13	ASTN2
rs10047326	10	A	C	0,0165	0,0026	1.263e-10	PIP4K2A
rs10509746	10	C	T	0,0269	0,0025	1.943e-26	Y_RNA
rs10821713	10	T	C	0,0168	0,0026	5.068e-11	ANK3
rs11012712	10	C	T	0,0223	0,0032	3.752e-12	
rs116454156	10	A	G	0,0778	0,0101	1.615e-14	FFAR4
rs12244851	10	T	C	0,0153	0,0027	1.450e-08	TCF7L2
rs1832007	10	G	A	0,0567	0,0035	6.779e-60	AKR1C4
rs2274224	10	G	C	0,0237	0,0025	1.017e-20	PLCE1
rs2801482	10	G	A	0,0501	0,0082	1.130e-09	CAMK1D
rs293275	10	C	T	0,0142	0,0025	1.603e-08	PRKG1
rs3858325	10	T	C	0,0186	0,0025	1.713e-13	GFRA1
rs4418728	10	G	T	0,0237	0,0025	5.991e-21	CYP26A1

rs4917962	10	T	G	0,0240	0,0039	5.216e-10	NOLC1
rs7910087	10	T	C	0,0175	0,0025	6.229e-12	C10orf11
rs7921105	10	C	T	0,0164	0,0026	1.962e-10	BEND7
rs9630085	10	G	A	0,0224	0,0031	9.144e-13	FFAR4
rs1039481	11	G	A	0,0418	0,0029	4.113e-48	PTPRJ
rs10767874	11	A	G	0,0145	0,0026	3.973e-08	DCDC1
rs10892564	11	G	A	0,0170	0,0026	4.611e-11	ARHGEF12
rs10893499	11	G	A	0,0218	0,0037	3.842e-09	ST3GAL4
rs11024614	11	C	T	0,0227	0,0026	3.409e-18	HPS5
rs11029620	11	C	T	0,0221	0,0031	4.620e-13	NUP98
rs11031058	11	T	C	0,0218	0,0033	5.389e-11	RPL12P30
rs117104648	11	C	T	0,0364	0,0052	3.240e-12	AP5B1
rs117600498	11	C	T	0,0379	0,0066	9.548e-09	ASCL2
rs12790261	11	A	C	0,0309	0,0046	1.211e-11	KDM2A
rs146345029	11	A	G	0,0342	0,0061	1.886e-08	GIF
rs174554	11	A	G	0,0221	0,0026	7.202e-17	FADS1
rs2512525	11	T	C	0,0240	0,0034	1.427e-12	USP35
rs3213223	11	A	G	0,0761	0,0030	9.35e-144	IGF2
rs34452566	11	T	G	0,0180	0,0031	1.032e-08	RP11-587D21.4
rs35023999	11	C	A	0,0153	0,0025	1.160e-09	ANKK1
rs4936759	11	C	T	0,0162	0,0025	1.949e-10	C11orf63
rs4980661	11	A	G	0,0145	0,0025	8.689e-09	AP000439.3
rs61867536	11	T	C	0,0179	0,0025	1.132e-12	MOB2
rs61904289	11	T	C	0,0164	0,0027	1.392e-09	EED
rs625245	11	G	T	0,0158	0,0027	4.364e-09	MRE11
rs6485702	11	T	C	0,0170	0,0027	1.415e-10	LRP4
rs67257872	11	A	G	0,0141	0,0025	2.315e-08	STK33
rs7115466	11	A	G	0,0155	0,0028	2.286e-08	H19
rs72858776	11	G	T	0,0299	0,0047	1.479e-10	RP11-396O20.2

rs7947951	11	G	A	0,0202	0,0027	1.025e-13	ARNTL
rs10745954	12	G	A	0,0151	0,0025	1.690e-09	RP11-328J6.1
rs10777540	12	G	T	0,0177	0,0025	2.236e-12	CRADD
rs10841649	12	C	T	0,0213	0,0037	9.254e-09	SLCO1B3
rs10860237	12	A	G	0,0301	0,0027	2.391e-28	RP11-1016B18.1
rs11064536	12	T	C	0,0199	0,0034	2.941e-09	WNK1
rs11111274	12	G	A	0,0799	0,0028	7.59e-175	IGF1
rs11175935	12	G	T	0,0200	0,0032	5.189e-10	LRRK2
rs117564283	12	T	C	0,0291	0,0050	5.046e-09	ACVRL1
rs12231073	12	T	G	0,0174	0,0025	5.421e-12	RNA5SP358
rs12425869	12	A	G	0,0183	0,0031	3.957e-09	SOCS2
rs1351394	12	C	T	0,0236	0,0025	6.924e-21	HMGA2
rs1800574	12	T	C	0,1446	0,0074	3.641e-84	HNF1A
rs2230281	12	A	G	0,0164	0,0028	4.010e-09	GALNT4
rs2460488	12	G	A	0,0257	0,0033	1.096e-14	RP11-110L15.1
rs247917	12	T	C	0,0152	0,0025	1.322e-09	ARID2
rs2657879	12	A	G	0,0199	0,0033	9.621e-10	GLS2
rs2856321	12	G	A	0,0261	0,0026	1.583e-23	ETV6
rs3759302	12	A	T	0,0206	0,0032	6.949e-11	KIAA1551
rs4547160	12	T	G	0,0177	0,0027	3.168e-11	AVPR1A
rs7314285	12	G	T	0,0520	0,0050	2.043e-25	CUX2
rs75938105	12	T	C	0,0467	0,0068	7.468e-12	RP11-110L15.2
rs773116	12	G	A	0,0162	0,0025	1.265e-10	ERBB3
rs78607331	12	C	T	0,0374	0,0060	6.059e-10	R3HDM2
rs9738365	12	A	C	0,0581	0,0028	1.905e-92	RP11-428G5.4
rs1170158	13	T	G	0,0210	0,0033	1.041e-10	DGKH
rs118081390	13	G	A	0,0278	0,0049	1.353e-08	FNDC3A
rs1535793	13	A	G	0,0237	0,0028	7.422e-17	LRCH1
rs6602909	13	C	T	0,0203	0,0027	4.354e-14	GAS6

rs71432868	13	C	T	0,0275	0,0050	3.105e-08	SNORA25
rs7323205	13	C	T	0,0148	0,0026	9.354e-09	LINC00676
rs9532512	13	A	G	0,0428	0,0032	8.024e-40	LINC00598
rs9573360	13	A	C	0,0143	0,0025	1.597e-08	LINC00402
rs9583151	13	C	T	0,0140	0,0025	2.708e-08	AL354741.1
rs10136874	14	G	T	0,0233	0,0025	2.350e-20	DLK1
rs10145154	14	T	C	0,0185	0,0030	9.924e-10	NRXN3
rs1061638	14	G	A	0,0177	0,0027	7.484e-11	AHSA1
rs1115897	14	A	C	0,0209	0,0027	1.540e-14	UNC79
rs13379043	14	T	C	0,0246	0,0029	5.776e-18	ELMSAN1
rs168961	14	G	A	0,0184	0,0025	2.889e-13	ZFP36L1
rs17106640	14	G	A	0,0170	0,0026	8.431e-11	ACTN1
rs175043	14	G	A	0,0178	0,0025	1.834e-12	EIF2B2
rs28396553	14	T	C	0,0152	0,0026	2.962e-09	AL162511.1
rs28929474	14	C	T	0,0631	0,0089	1.294e-12	SERPINA1
rs33912345	14	C	A	0,0232	0,0026	2.363e-19	SIX6
rs36215895	14	C	T	0,0819	0,0129	2.121e-10	SYNE2
rs78598185	14	G	A	0,0288	0,0044	7.090e-11	SLC24A4
rs79936318	14	A	G	0,0173	0,0032	3.875e-08	SYNE2
rs8017377	14	G	A	0,0167	0,0025	3.353e-11	NYNRIN
rs11856160	15	A	G	0,0207	0,0035	2.843e-09	CHD2
rs12442867	15	A	C	0,0167	0,0026	9.329e-11	RP11-299H22.1
rs12593755	15	T	G	0,0155	0,0026	1.846e-09	RP11-97O12.3
rs12912439	15	T	C	0,0219	0,0027	1.246e-15	LINC01197
rs142354201	15	G	A	0,0340	0,0058	3.677e-09	PGPEP1L
rs17747633	15	G	A	0,0150	0,0025	3.080e-09	KNL1
rs2004839	15	G	A	0,0210	0,0034	4.336e-10	RP11-299H22.3
rs2311313	15	G	T	0,0189	0,0033	1.691e-08	RP11-35O15.1
rs2930313	15	G	A	0,0276	0,0047	4.141e-09	CCDC33

rs4545755	15	G	A	0,0159	0,0025	3.865e-10	CYP19A1
rs55707100	15	C	T	0,1507	0,0081	1.406e-76	MAP1A
rs5742915	15	C	T	0,0246	0,0025	1.723e-22	PML
rs79076440	15	A	G	0,0189	0,0033	1.164e-08	USP3
rs8033075	15	A	G	0,0448	0,0052	1.061e-17	PIAS1
rs11077337	16	T	G	0,0154	0,0025	1.454e-09	ZNF597
rs11149612	16	C	T	0,0271	0,0025	1.131e-26	RP11-505K9.4
rs111792934	16	C	T	0,0216	0,0034	1.430e-10	HAS3
rs116971887	16	G	T	0,0359	0,0061	4.895e-09	SALL1
rs12597502	16	G	A	0,0150	0,0027	4.528e-08	CHD9
rs12927172	16	A	G	0,0153	0,0026	3.066e-09	IL4R
rs12935091	16	G	A	0,0349	0,0058	2.235e-09	ZNF19
rs12935465	16	T	C	0,0165	0,0025	5.769e-11	XYLT1
rs143076454	16	G	A	0,0719	0,0093	1.245e-14	LMF1
rs147491123	16	C	T	0,0360	0,0065	3.286e-08	LINC01572
rs1532824	16	A	C	0,0169	0,0029	4.521e-09	ATF7IP2
rs1548917	16	T	C	0,0152	0,0025	2.361e-09	RP11-461O7.1
rs1657125	16	T	G	0,0321	0,0037	2.007e-18	MEIOB
rs17299478	16	C	T	0,0319	0,0034	2.497e-21	NOB1
rs2023762	16	T	C	0,0147	0,0025	5.991e-09	SYT17
rs4786350	16	C	G	0,0361	0,0062	6.048e-09	IFT140
rs4788220	16	G	A	0,0174	0,0025	5.263e-12	FAM57B
rs4985062	16	T	C	0,0152	0,0025	2.040e-09	USP7
rs4988483	16	C	A	0,1720	0,0056	1.87e-203	SSTR5
rs61731445	16	C	T	0,0283	0,0050	1.958e-08	
rs7204824	16	C	T	0,0240	0,0029	1.168e-16	LMF1
rs72761177	16	A	G	0,0775	0,0044	7.205e-70	NUBP2
rs74774288	16	G	T	0,0274	0,0032	2.955e-17	RP11-420N3.3
rs7498665	16	G	A	0,0193	0,0026	7.173e-14	SH2B1

rs750952	16	C	T	0,0318	0,0026	1.105e-34	ZNF646
rs753108	16	A	G	0,0247	0,0029	1.606e-17	CMIP
rs80253441	16	T	C	0,1313	0,0105	8.227e-36	IGFALS
rs8054054	16	G	A	0,0153	0,0025	1.026e-09	CCDC154
rs8054322	16	A	G	0,0149	0,0025	4.723e-09	GSE1
rs8059803	16	A	G	0,0307	0,0027	4.415e-29	CMIP
rs8182173	16	T	C	0,0176	0,0029	1.610e-09	CORO7-PAM16
rs142377191	17	A	G	0,1254	0,0088	2.048e-46	DCAF7
rs1801689	17	C	A	0,0894	0,0074	1.248e-33	APOH
rs199525	17	G	T	0,0200	0,0030	5.613e-11	WNT3
rs2309401	17	T	G	0,0149	0,0026	6.785e-09	NLRP1
rs35819807	17	T	C	0,0174	0,0029	2.387e-09	KCNH6
rs3760237	17	C	T	0,0241	0,0025	1.441e-21	SCN4A
rs4075483	17	C	T	0,0166	0,0026	1.312e-10	BAIAP2
rs4789227	17	T	C	0,0155	0,0026	4.335e-09	UNK
rs56030650	17	C	A	0,0220	0,0025	2.417e-18	GSDMA
rs6416868	17	A	G	0,0193	0,0025	3.115e-14	TTC19
rs6501601	17	G	A	0,0152	0,0026	4.071e-09	POLR3KP2
rs668799	17	C	T	0,0175	0,0028	4.019e-10	COASY
rs7502910	17	A	G	0,0161	0,0025	1.528e-10	WDR81
rs76708468	17	C	T	0,0873	0,0065	3.084e-41	1ERN
rs77542162	17	G	A	0,0543	0,0086	2.412e-10	ABCA6
rs8075153	17	C	T	0,0215	0,0025	1.831e-17	RAI1
rs8079923	17	C	T	0,0157	0,0029	3.664e-08	AKAP10
rs9892862	17	G	A	0,0224	0,0030	9.705e-14	Y_RNA
rs11152071	18	C	T	0,0200	0,0029	8.957e-12	RP11-1151B14.2
rs12454712	18	T	C	0,0180	0,0026	4.051e-12	BCL2
rs190102446	18	C	T	0,0412	0,0069	2.452e-09	RP11-27G24.1
rs57551555	18	T	G	0,0189	0,0026	5.817e-13	RP11-176N18.2

rs585187	18	T	G	0,0148	0,0025	3.712e-09	MRPS5P4
rs8084351	18	G	A	0,0154	0,0025	8.537e-10	DCC
rs8095538	18	G	T	0,0205	0,0027	8.447e-14	-
rs8097893	18	A	G	0,0577	0,0062	1.723e-20	GALR1
rs11671304	19	C	T	0,0176	0,0027	6.884e-11	ZC3H4
rs12975366	19	T	C	0,0204	0,0026	2.253e-15	LILRB5
rs2287922	19	G	A	0,0305	0,0025	1.833e-33	RASIP1
rs296361	19	A	G	0,0247	0,0034	3.421e-13	SULT2A1
rs34536443	19	G	C	0,0446	0,0060	9.964e-14	TYK2
rs3760954	19	T	C	0,0235	0,0037	2.909e-10	MIR7-3HG
rs58560372	19	C	T	0,0198	0,0035	1.331e-08	SPINT2
rs58658292	19	G	A	0,0305	0,0056	4.618e-08	ZNF536
rs62102136	19	C	T	0,0155	0,0027	6.527e-09	LSM14A
rs6510033	19	A	G	0,0201	0,0028	1.147e-12	AC005597.1
rs6510177	19	T	C	0,0227	0,0032	1.598e-12	ZNF536
rs6510832	19	G	T	0,0334	0,0053	2.638e-10	KDM4B
rs67868323	19	T	G	0,0158	0,0028	2.205e-08	ZBTB7A
rs7254601	19	G	A	0,0164	0,0029	2.551e-08	COX6B1
rs7256521	19	G	A	0,0155	0,0025	9.761e-10	ZNF845
rs8105174	19	C	T	0,0497	0,0032	7.971e-54	DNMT1
rs8112883	19	G	T	0,0169	0,0028	1.166e-09	INSR
rs8113618	19	T	C	0,0307	0,0025	1.185e-33	QTRT1
rs16995311	20	A	C	0,0396	0,0046	5.559e-18	PTPN1
rs17265513	20	T	C	0,0219	0,0031	3.454e-12	ZHX3
rs2207132	20	G	A	0,0477	0,0070	1.064e-11	LINC01728
rs2424396	20	G	A	0,0329	0,0043	2.245e-14	LINC01726
rs2738787	20	A	G	0,0366	0,0046	1.251e-15	TNFRSF6B
rs4809401	20	T	C	0,0235	0,0038	6.488e-10	NPBWR2
rs6037508	20	G	T	0,0173	0,0030	8.740e-09	SLC4A11

rs6046825	20	A	C	0,0240	0,0028	2.108e-17	RALGAPA2
rs6088579	20	G	A	0,0272	0,0033	8.509e-17	NCOA6
rs7267595	20	A	C	0,0155	0,0025	9.437e-10	JAG1
rs7508949	20	C	G	0,0248	0,0025	1.490e-22	CRNKL1
rs75989562	20	A	G	0,0303	0,0053	8.093e-09	APMAP
rs9978775	21	G	A	0,0186	0,0025	2.351e-13	BRWD1-AS1
rs12106594	22	T	C	0,0357	0,0059	1.588e-09	EIF4ENIF1
rs2412973	22	A	C	0,0140	0,0025	3.334e-08	HORMAD2
rs4823324	22	T	C	0,0164	0,0026	1.938e-10	ATXN10
rs5755948	22	A	G	0,0275	0,0036	4.375e-14	RBFOX2
rs6519133	22	T	C	0,0292	0,0026	7.731e-30	JOSD1
rs8138950	22	C	T	0,0153	0,0025	1.323e-09	ZNRF3
rs9611565	22	T	C	0,0290	0,0029	3.825e-23	TEF

Chr, chromosome; EA, effect allele; NEA, non-effect allele; SNP, single-nucleotide polymorphisms.

Table S2. Sources and definition of cancers in UK Biobank

Cancer site	ICD-9 codes	ICD-10 codes	Self-reported cancer
Prostate	185, V10.46	C61, Z85.46	1044
Breast	174, 175, V10.3	C50, Z85.3	1002
Ovary	183.0, 183.2, 183.8, 183.9, V10.43	C56, C57.0, C57.4, Z85.43	1039
Uterus	179, 182, V10.42,	C54, C55, Z85.42	1040
Cervix	180, V10.41	C53, Z85.41	1041
Bladder	188, 189.1, 189.2, V10.51, V10.53	C67, C65, C66, Z85.51, Z85.54, Z85.53	1035
Kidney	189.0, V10.52	C64, Z85.528	1034
Head/neck	140, 141, 142, 143, 144, 145, 146, 147, 148, 149, 160, 161, V10.01, V10.02, V10.21, V10.22	C00, C01, C02, C03, C04, C05, C06, C07, C08, C09, C10, C11, C12, C13, C14, C30, C31, C32, Z85.21, Z85.22, Z85.81	1006, 1007, 1009, 1004, 1010, 1011, 1012, 1077, 1078, 1079, 1005, 1015, 1016
Colorectum	153, 154.0, 154.1, V10.05, V10.06	C18, C19, C20, Z85.038, Z85.048	1020, 1022, 1023
Pancreas	157	C25, Z85.07	1034
Melanoma	172, V10.82	C43, Z85.820	1059
Leukaemia	204, 205, 206, 207, 208, V10.6	C91, C92, C93, C94.0, C94.2, C94.3, C94.4, C94.8, C95, Z85.6	1048, 1055, 1056, 1074
NHL	200, 202.0, 202.1, 202.2, 202.7, V10.71	C82, C83, C84, C85, C86, C88.0, C88.4, Z85.72	1053
Lung	162, V10.1	C33, C34, C39.9, Z85.1	1001, 1027, 1028, 1080

Table S3. Power calculation for the associations of insulin-like growth factor-1 with site-specific cancers

				Power to detect OR of the following magnitude							
**Cancer site**	**Data source**	**Cases**	**Sample size**	**0.70**	**0.80**	**0.90**	**0.95**	**1.05**	**1.10**	**1.20**	**1.30**
Prostate	PRACTICAL	79 148	140 254	1.00	1.00	1.00	0.83	0.79	1.00	1.00	1.00
Breast	BCAC	122 977	228 951	1.00	1.00	1.00	0.96	0.95	1.00	1.00	1.00
Ovary	OCAC	25 509	66 450	1.00	1.00	0.98	0.50	0.47	0.96	1.00	1.00
Lung	ILCCO	11 348	27 209	1.00	1.00	0.74	0.25	0.23	0.76	1.00	1.00
Prostate	UK Biobank	7872	168 748	1.00	1.00	0.83	0.30	0.30	0.82	1.00	1.00
Breast	UK Biobank	13 666	198 838	1.00	1.00	0.94	0.41	0.41	0.93	1.00	1.00
Ovary	UK Biobank	1520	198 838	0.95	0.66	0.22	0.09	0.09	0.22	0.66	0.94
Uterus	UK Biobank	1931	198 838	0.98	0.77	0.27	0.10	0.10	0.27	0.76	0.98
Cervix	UK Biobank	1928	198 838	0.98	0.77	0.27	0.10	0.10	0.27	0.76	0.98
Bladder	UK Biobank	2588	367 586	1.00	0.87	0.34	0.12	0.06	0.06	0.87	1.00
Kidney	UK Biobank	1310	367 586	0.91	0.60	0.20	0.09	0.09	0.20	0.60	0.91
Head-neck	UK Biobank	1615	367 586	0.96	0.69	0.23	0.09	0.09	0.23	0.69	0.96
Colorectum	UK Biobank	5486	367 586	1.00	0.99	0.62	0.20	0.20	0.62	0.99	1.00
Pancreas	UK Biobank	1264	367 586	0.91	0.60	0.20	0.09	0.09	0.20	0.60	0.91
Melanoma	UK Biobank	4869	367 586	1.00	0.99	0.57	0.19	0.19	0.57	0.99	1.00
Leukemia	UK Biobank	1403	367 586	0.93	0.63	0.21	0.09	0.09	0.21	0.63	0.93
Non-Hodgkin lymphoma	UK Biobank	2296	367 586	0.99	0.83	0.31	0.11	0.11	0.31	0.83	0.99
Lung	UK Biobank	2838	367 586	1.00	0.90	0.37	0.13	0.13	0.37	0.99	1.00
Prostate	BioBank Japan	5408	109 347	1.00	0.99	0.60	0.20	0.20	0.59	0.99	1.00
Lung	BioBank Japan	4050	212 453	1.00	0.97	0.49	0.16	0.16	0.49	0.97	1.00
Colorectum	BioBank Japan	7062	202 807	1.00	1.00	0.72	0.25	0.25	0.71	1.00	1.00
Esophagus	BioBank Japan	1300	197 045	0.91	0.60	0.20	0.09	0.09	0.09	0.60	0.91
Stomach	BioBank Japan	6563	202 308	1.00	1.00	0.69	0.23	0.23	0.69	1.00	1.00
Liver	BioBank Japan	1866	197 611	0.98	0.75	0.26	0.10	0.10	0.26	0.75	0.98

Table S4. Associations of genetically-predicted insulin-like growth factor-1 levels with colorectal, prostate and breast cancer in multivariable Mendelian randomization analyses adjusting for height

Cancer site	Data source	Cases	Sample size	OR	95% CI	P
Colorectum	UK Biobank	5486	367 586	1.10	1.00-1.21	0.06
Prostate	PRACTICAL	79 148	140 254	1.03	0.97-1.10	0.04
Prostate	UK Biobank	7872	168 748	1.11	1.00-1.22	0.04
Breast	BCAC	122 977	228 951	1.07	1.02-1.13	0.01
Breast	UK Biobank	13 666	198 838	0.98	0.91-1.06	0.66

BCAC, Breast Cancer Association Consortium; CI, confidence interval; OR, odds ratio; PRACTICAL, Prostate Cancer Association Group to Investigate Cancer Associated Alterations in the Genome consortium.

Table S5. Associations of genetically-predicted insulin-like growth factor-1 levels with site-specific cancers in sensitivity analyses

			Weighted median		MR-Egger estimate			MR-Egger intercept		Contamination mixture		MR-PRESSO			
**Cancer site**	**Data source**	**Cases**	**OR (95% CI)**	***P***	**OR (95% CI)**	***P***	**Rücker's Q**	**Intercept**	***P***	**OR (95% CI)**	***P***	**Outliers**	**OR (95% CI)***	***P***	**Distortion test, *P***
Colorectum	UK Biobank	5486	1.21 (1.04-1.40)	0.01	1.20 (0.98-1.47)	0.08	482.4	-0.002 (-0.007 to 0.003)	0.40	1.34 (1.16-1.52)	8.4E-5	0	1.11 (1.01-1.22)	0.03	NA
Prostate	PRACTICAL	79 148	1.03 (0.97-1.09)	0.95	0.89 (0.78-1.03)	0.11	896.4	0.004 (0.000 to 0.007)	0.03	0.98 (0.91-1.07)	0.50	7	1.03 (0.97-1.08)	0.36	0.991
Prostate	UK Biobank	7872	1.09 (0.96-1.24)	0.20	1.13 (0.92-1.38)	0.25	629.8	0.000 (-0.006 to 0.005)	0.82	1.11 (0.99-1.28)	0.08	3	1.10 (1.01-1.20)	0.04	0.919
Breast	BCAC	122 977	1.10 (1.05-1.17)	<0.001	1.09 (0.97-1.23)	0.15	1087.8	0.000 (-0.003 to 0.003)	0.80	1.17 (1.06-1.21)	4.8E-4	12	1.09 (1.04-1.14)	5.7E-5	0.581
Breast	UK Biobank	13 666	1.00 (0.90-1.10)	0.94	0.92 (0.79-1.07)	0.30	650.4	0.002 (-0.002 to 0.006)	0.28	0.89 (0.83-0.96)	0.01	5	0.99 (0.93-1.06)	0.84	0.996
Ovary	OCAC	25 509	1.01 (0.91-1.13)	0.80	1.02 (0.85-1.21)	0.86	548.7	-0.002 (-0.006 to 0.002)	0.39	0.99 (0.90-1.12)	0.78	1	0.99 (0.87-1.01)	0.10	0.841
Ovary	UK Biobank	1520	0.99 (0.75-1.30)	0.95	1.10 (0.77-1.57)	0.61	407.2	-0.005 (-0.014 to 0.004)	0.27	0.88 (0.71-1.17)	0.45	0	0.92 (0.78-1.08)	0.31	NA
Lung	ILCCO	11 348	1.04 (0.91-1.21)	0.55	1.15 (0.92-1.44)	0.22	518.6	-0.003 (-0.009 to 0.002)	0.23	1.12 (0.94-1.30)	0.18	1	1.03 (0.93-1.13)	0.59	0.899
Lung	UK Biobank	2838	1.19 (0.97-1.45)	0.10	1.51 (1.14-1.99)	0.004	469.1	-0.009 (-0.016 to -0.002)	0.01	1.11 (0.90-1.39)	0.37	1	1.08 (0.95-1.23)	0.23	0.884
Uterus	UK Biobank	1931	1.15 (0.89-1.47)	0.29	1.11 (0.78-1.57)	0.56	496.8	-0.002 (-0.011 to 0.007)	0.60	1.09 (0.90-1.42)	0.32	0	1.02 (0.87-1.20)	0.80	NA
Cervix	UK Biobank	1928	0.99 (0.78-1.27)	0.96	1.09 (0.77-1.54)	0.64	499.7	-0.003 (-0.012 to 0.006)	0.55	1.17 (0.88-1.52)	0.22	1	1.00 (0.85-1.17)	0.99	0.003
Bladder	UK Biobank	2588	1.17 (0.95-1.45)	0.14	1.13 (0.84-1.52)	0.43	489.5	-0.004 (-0.012 to 0.003)	0.26	1.17 (0.93.1.43)	0.19	0	0.97 (0.84-1.11)	0.64	NA
Kidney	UK Biobank	1310	0.99 (0.73-1.34)	0.93	1.03 (0.70-1.54)	0.87	440.7	-0.000 (-0.010 to 0.100)	0.97	1.01 (0.73-1.35)	0.91	0	1.03 (0.85-1.23)	0.78	NA
Head/neck	UK Biobank	1615	1.04 (0.79-1.35)	0.80	1.02 (0.72-1.44)	0.90	383.3	0.000 (-0.009 to 0.009)	0.98	0.83 (0.66-1.11)	0.23	0	1.02 (0.87-1.19)	0.83	NA
Pancreas	UK Biobank	1264	0.88 (0.65-1.19)	0.40	1.27 (0.84-1.93)	0.26	469.6	-0.010 (-0.021 to 0.001)	0.06	0.84 (0.54-1.11)	0.22	0	0.89 (0.74-1.08)	0.83	NA
Melanoma	UK Biobank	4869	0.99 (0.85-1.16)	0.90	1.09 (0.87-1.36)	0.72	522.7	-0.002 (-0.009 to 0.003)	0.34	0.90 (0.79-1.14)	0.64	2	1.01 (0.91-1.11)	0.89	0.08
Leukaemia	UK Biobank	1403	1.07 (0.81-1.43)	0.63	0.94 (0.65-1.36)	0.75	407.9	0.000 (-0.010 to 0.009)	0.87	0.89 (0.67-1.25)	0.34	0	0.91 (0.77-1.09)	0.31	NA
NHL	UK Biobank	2296	1.01 (0.80-1.26)	0.96	1.10 (0.81-1.48)	0.55	445.0	-0.002 (-0.010 to 0.005)	0.54	0.71 (0.59-0.94)	0.01	0	1.01 (0.88-1.16)	0.93	NA

*Outlier corrected results. If no outliers were identified, results are the inverse-variance weighted estimate (same as in Figure 1). BCAC, Breast Cancer Association Consortium; CI, confidence interval; ILCCO, International Lung Cancer Consortium; MR, Mendelian randomisation; NHL; Non-Hodgkin lymphoma; OCAC, Ovarian Cancer Association Consortium; PRACTICAL, Prostate Cancer Association Group to Investigate Cancer Associated Alterations in the Genome consortium.

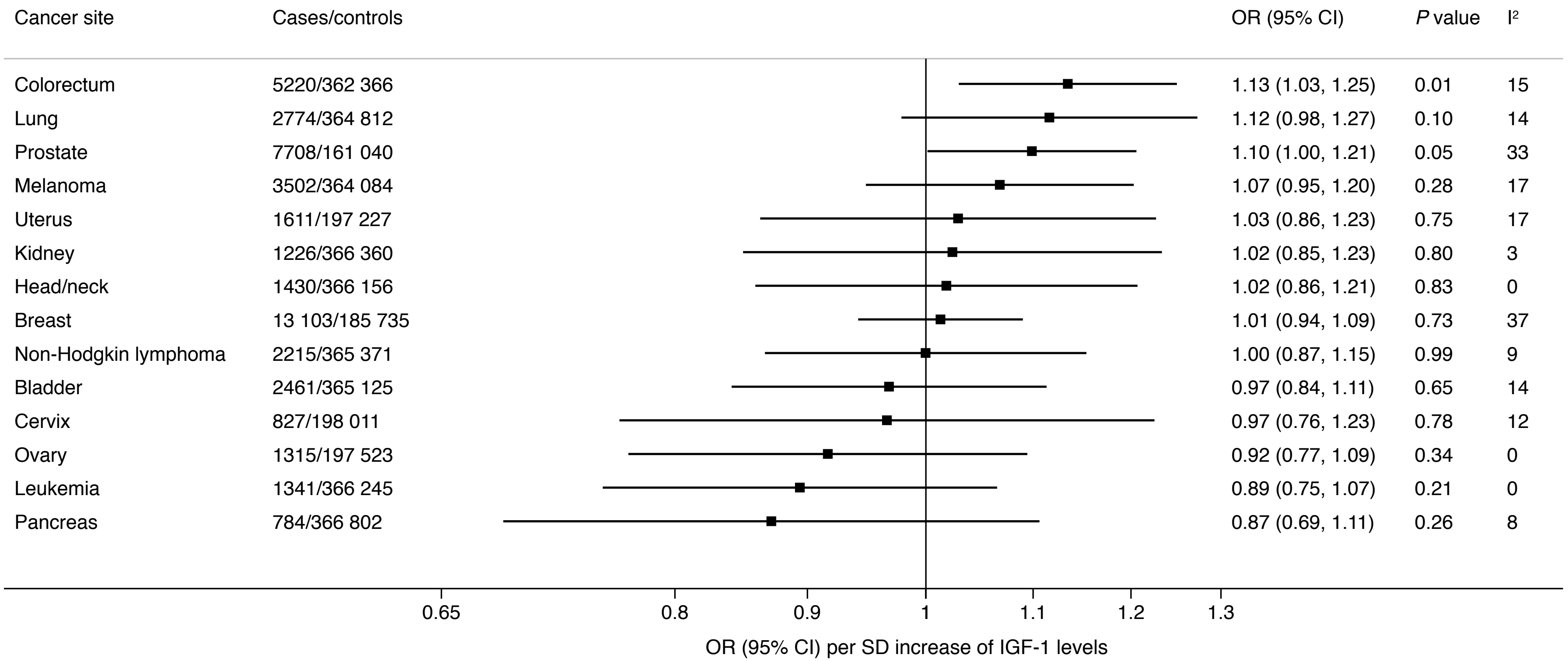

Figure S1. Associations of genetically predicted serum IGF-1 levels with site-specific cancers in European-descent individuals in UK Biobank in a sensitivity analysis excluding self-reported cancer. Estimates were derived using the multiplicative random-effects inverse-variance weighted method and were based on up to 416 single-nucleotide polymorphisms associated with IGF-1 levels at the genome-wide significance threshold. I^2 value is a measure of heterogeneity among estimates from individual single-nucleotide polymorphisms.
